# Pain and temporality: a merleau-pontyian approach

**DOI:** 10.1007/s11019-024-10205-y

**Published:** 2024-05-18

**Authors:** Judith N Wagner

**Affiliations:** 1 Faculty of Humanities, Department of Philosophy, Charles University, Prague, Czech Republic; 2 Evangelisches Klinikum Gelsenkirchen, Teaching Hospital University Duisburg-Essen, Munckelstrasse 27, 45879 Gelsenkirchen, Germany

**Keywords:** Pain, Temporality, Lived body, Enactivism, Phenomenology

## Abstract

Chronic pain is a common disorder with enormous sociomedical importance. A major part of primary and secondary costs of illness is caused by the various pain syndromes. Nociception – the sensory perception of a painful stimulus – is a complex process relying on an intricate system of anatomical, neurophysiological and biochemical networks. This applies even more so to pain – the state of experiencing a nociceptive event, of interpreting it in terms of meaning for the affected individual and of suffering a range of emotions it elicits. This intricacy renders it obvious, that the empirical medical sciences alone cannot explain all aspects of pain. Hence, it has also become a focus of phenomenological research. One aspect of these investigations is the interaction of pain and the perception of the lived body’s spatiality. The focus of this article will build on these concepts to develop a construct of the alteration of temporality caused by chronic pain and the effects this spells out for the affected subject. To this end, I will primarily draw on Merleau-Ponty’s ideas of the lived body as well as on theories of enactivism and embodiment. I will also point out parallels to neuroscientific data, thereby demonstrating the proximity of phenomenology and neuroscience. A possible partial solution to the pain dilemma may be derived from psychology: techniques relying on cognitive behavioural intervention, awareness training, and existential analysis may provide alleviation to patients suffering from chronic pain.

## Introduction

Chronic pain syndromes constitute an important social and medical challenge: they amounted to up to $635 billion in medical expenses in the US in 2008 – more than those caused by heart disease, cancer, or diabetes (Gaskin & Richard 2012).^1^ On the other side, the medical infrastructure providing for patients with pain – particularly chronic pain – syndromes is ever expanding. However, a continuing undersupply is being claimed.^2^ Would a further increase in pain-specific medical facilities assuage the problem? Or might it even exacerbate it, for example by iatrogenic fixation on pain killers and invasive procedures? This article aims at providing a foundation for a more holistic, interdisciplinary discussion of the complex phenomenon „pain“.

While philosophy and neurosciences have experienced a somewhat conflictive relationship for a long time, recent years have seen multiple attempts to unite the two perspectives and have emphasized the importance of the multidisciplinary approach. Phenomenology is an attempt to understand the primary conditions of human lived experience by describing how its objects, the phenomena, appear to the subject without resorting to a priori theoretical constructs. It is a philosophical technique very much suited for the description of the lived human body in health and disease and has been applied to pursuits as varied as elucidating the general nature of patient autonomy and the norms of clinical decision making (Lewis & Holm 2022, Oh 2021), the experience of aging (Van Rhyn et al. 2020), and establishing a phenomenological framework for conditions as different as congenital illness (McConville 2021), erectile dysfunction (Suijker et al. 2021), COVID-19 infection (Missel et al. 2022), obesity and other eating disorders (Juli& Juli 2020; Natvik & Råheim 2021, Haga et al. 2020), depression and burn-out (Slatman & van de Ven 2021, Engebretsen et al. 2020), limb-threatening ischemia (Monaro et al. 2020), cancer (Yang et al. 2021), as well as specific interventions such as dance and physiotherapy (Purser 2021; Halák & Kříž 2022).

Merleau-Ponty is an important representative of the phenomenological school of thought who focused on the lived body as the correlate of perception. His thoughts have in particular been called upon to draft more holistic constructs of disease and disability (Edwards 1998), with a particular interest on the changing quality of sensory perception in states of disrupted health, including chronic pain syndromes (Pedersen et al. 2021, (Valenzuela-Moguillansky et al. 2017). A particular focus in this context has been on the spatiality of the human body. In their exploration of phantom pain, Nortvedt & Engelsrud point out that patients report a transgression of their bodily limits („feeling invaded by insects“; Nortvedt & Engelsrud 2014), while Valenzuela-Moguillansky et al. report on fibromyalgia patients perceiveing the affected body part or the entire body to be enlarged, while experiencing their peripersonal space (space that is defined to be at arm’s length) to constrict.

The aim of this paper is to propose a construct on the temporality of bodily perception and how it is altered by chronic pain. In order to do so, I will draw heavily on phenomenological theories, foremost by Merleau-Ponty. However, I will also integrate neuroscientific findings, paralleling Merleau-Ponty’s approach to demonstrate that philosophy and empirical sciences do not oppose but rather inform each other.

First of all, I briefly summarize the phenomenological distinction between the lived body and the body as an object. As there are close parallels between how we perceive our lived body in terms of temporality and spatiality, I will add some ideas on how pain interferes with the perceived boundaries of the lived body. In order to be able to discuss the contribution of this paper to the topic, I will continue with a review on the current status of the phenomenological debate on temporality and its effects on the perception of pain.

## Phenomenology: the lived body and its being-to-the-world

Phenomenology is the study of human experience and of how objects, subjects, sensations, emotions, and abstract concepts present themselves to us. With his “Ideas”, Edmund Husserl is traditionally seen as the founder of this philosophical technique (Gallagher 2012; p. 7). The phenomenology of embodiment is very much associated with Husserl’s concept of the human body, which he considered a unique entity due to its ambiguous quality. On the one hand it is material and can as such be perceived from the out- and from the inside. On the other hand – and simultaneously – it serves as the subject’s means of experience while freely moving into its environment. As the „center of orientation“, it is the pivotal point of motion and perception. As such, it relates the external world to the subject (Husserl 1989, 165–169). This view of the lived body (Leib) is that of embodied subjectivity, of a third entity overcoming the dualism between the mental and the physical, of the material and the spiritual, of the subject’s in- and outside. It illustrates the singularity of the human body, but also heralds its vulnerability when the lived body is confronted and burdened with its materiality. As such, the body not only “is”, it also “feels like” something. It is this characteristic that allows for perceptions to alter and modulate the bodily sensation.

Merleau-Ponty elaborates on Husserl’s notion of the body and introduces neurosciences and psychology to complement it. The lived body is the subject’s means of perception, at the same time as it allows her to move into and to intentionally interact with her world. Hence, it is the lived body that interweaves the physical body, cognition, and the objective as well as the interpersonal environment, integrating the subject into her ecological niche: “The body is the vehicle of being in the world and, for a living being, having a body means being united with a definite milieu, merging with certain projects, and being perpetually engaged therein” (Merleau-Ponty 2013; p. 84). Within this interaction, the lived body can be described as a spatial continuum whose perceived boundaries are not fixed but modifiable.

With this in mind, many phenomenological constructs of the body in pain have focused on the idea of spatiality. A painful body part may feel enlarged, making it the center of attention, becoming all-embracing. On the other hand, a (potentially) painful stimulus may cause the subject to withdraw, to minimize the point of contact and lead to a contraction of the peripersonal space (Dietrich 2015, 364-6).

Spatiality is closely related to materiality. The lived body is distinguished by the characteristics of permanence and transparency. While the material body is always present, it remains at the border of perception as long as it is well-functioning and healthy (Carel 2018; p. 77) – it is transparent, a vehicle enabling us to interact with our environment without becoming a focus of attention itself:

…bodily presence is of a highly paradoxical nature. While in one sense the body is the most abiding and inescapable presence in our lives, it is also essentially characterized by absence. That is, one’s own body is rarely the thematic object of experience (Leder 1990; p. 1).

This transparency is suspended once the lived body is afflicted by ‘limit-situations’ such as illness, handicap or unpleasant physiological perceptions. Pain is certainly one of these instances, leading to an accentuation of the body’s material facet, while the perception of the body as lived moves to the background, altering temporo-spatial and interpersonal perception. In pain, the focus is turned away from the outer environment and directed towards the aching site (Rysewyck, 2017, 175-6). The enforced withdrawal has a totalizing, all-encompassing effect on the individual, leading to estrangement and isolation from her environment, taking away the lightness and implicitness of moving in the world (Maio, 2015, 170-3). This in turn destroys the inherent trust in the body and its capacities to deal with the environmental challenges, giving rise to a perceived loss of control or ‚bodily doubt‘: „The body is a problem, an obstacle, a stranger. I never befriended that stranger” (Carel 2018, 87–96;116).^,^^3^

But not only does pain have an intimate nexus with spatiality, it also alters temporal perception, causing shifts of boundaries, appearances of hiatus, and blurring of time spans. Massive and / or constant pain entails an element of totalization:

Pain — has an Element of Blank —.

It cannot recollect.

When it begun — or if there were.

A time when it was not — (Emily Dickinson 2021).

Pain extinguishes a past that cannot be recalled as a time without pain and foreshadows a future that cannot be imagined in any other way than filled with ache. The person is stalled in the present: while the external time proceeds, the inner time has stopped.

## Pain and temporality

### The phenomenological perspective on temporality

Merleau-Ponty describes the subject as „…temporal, not through some accident of the human constitution, but in virtue of an inner necessity” (Merleau-Ponty 2013; p. 432). What does he mean by “inner necessity”? One of the best known contemporary phenomenological descriptions of temporality is Husserl’s concept of time as a retentional-protentional extension. He illustrates this idea by the example of hearing a melody: the perception of the ongoing tone mingles with the memory of immediately preceding moments (retention) as well as with the anticipation of its continuation (protention). Hence, the present moment does not have a punctiform, but rather an extended character. It is charged with meaning due to its embeddedness in the past and its orientation towards the future (Husserl 1964, Part One, Section One, § 3).

On this background, Merleau-Ponty stresses the tight embrace of the current moment by past and future, which prevents it from becoming arbitrary:

The present still holds in hand the immediate past, …. The same goes for the imminent future that will itself have its own horizon of imminence. But along with my immediate past, I also have the horizon of the future that surrounded it; that is, I have my actual present seen as the future of that past. Along with the imminent future, I also have the horizon of the past that will surround it; that is, I have my actual present as the past of that future (Merleau-Ponty 2013, 71 − 2).

Just as space is not a fixed entity, time is determined by the specific perspective of the individual and her relation to the objects in her world. In analogy to the unifying consciousness that relates the lived body to its individual parts – a synthesis that is primordially embodied rather than actively reflected upon – the subject’s perspective imparts cohesiveness on the unfolding of time (Olivier 2007, 15 − 7).

Through its position in the bracket of the double horizon of protention and retention, the lived moment can be anchored on a measurable trajectory of consecutive biographical events and is endowed with continuity (Merleau-Ponty 2013; p. 72). Just as the lived body is the pivot point of spatial perception, it is the reference for the unfolding time. The experience of the one-after-the-other elicits the perception of a continuous time course, of the extended moment: „Past and future exist all too well in the world, they exist in the present, …” (Merleau-Ponty 2013; p. 434). In contrast, the objective, measured time – Merleau-Ponty’s ‘universal time’ – represents an abstraction of this ‘lived time’ (Merleau-Ponty 2013; p. 73; Förster 2008; p. 4).

Time cannot be found within the objects themselves and neither is it located solely within the subject’s consciousness. Such an assumption would lead to an infinite regression: „…if the consciousness of time was built from successive states of consciousness, then a new consciousness would be necessary for the awareness of this succession, and so on” (Merleau-Ponty 2013; p. 446). Following Husserl, Merleau-Ponty postulates an originary consciousness, a pre-predicative form of temporality. This perception of time enables the subject to perceive herself as continuous:

I am not, for myself, directed toward the present time; I am just as much directed toward this morning or toward the night that is about to arrive, and although my present is surely this present instant, it is also just as much today, this year, or even my entire life. There is no need for a synthesis that would externally connect the tempora (…) into a single time, because each of these tempora already included, beyond itself, the open series of other tempora … (Merleau-Ponty 2013; p. 444).

Just as the individual structures her ecological niche in terms of space, she does so in terms of time:

Time presupposes a view upon time. … This metaphor (time as a flowing river, note from the author) has been able to survive since Heraclitus up until today because we surreptitiously place in the river a witness to its flowing (Merleau-Ponty 2013; pp. 433-4).

In a similar manner as Merleau-Ponty postulates a lived time and an abstract universal time, the protagonists of enactivism juxtapose an objective and a subjective time. Gallagher, for instance, distinguishes a measurable time – ‚timing’– from an intrinsic time – ‚temporality’ – as a preconscious, innate knowledge of a past and future sequence of movement (Gallagher 2011; p. 421). He illustrates this assumption by referring to data proving a meaningful coordination of hand movements and mouth opening in the fetus. This sequence anticipates food intake and suggests an inherent consciousness of ‘before’ and ‘after’, demonstrating the strong interconnection between the concepts of temporality, corporeality, body schema, and motor activity.

Hence, temporality and the subject mutually determine each other: just as the subject exists within a temporal frame, time exists due to the subject’s perspective on the flow of time. The individual does not only *have* time, she *exists* in time (Wehrle 2020; p. 506) within an embodied experience of temporality, which is constituted by her body’s movements with their inherent notion of the past and anticipation of the future: „…temporal constitution concretely takes place in the lived body’s actual performance of movements, which integrate, in turn, the dimensions of past, present and future by means of an intentional arc (…)” (Wehrle 2020; p. 508). It follows from this assumption that future experiences will be influenced by the lived moment – and hence indirectly by the preceding one.

### Pain and temporality – the state of the phenomenological discussion

The phenomenological consideration of the interaction between pain and temporality has traditionally focussed on two aspects. It has delineated the breaks in and the distortion of the lived time or temporality and the concomitant alienation of the sufferer from her objective and intersubjective environment. Furthermore, phenomenologists have investigated the influence that a present filled with pain has on the perception of the past and the future. This development started with Sartre’s description of time and its relevance for pain and illness. He defines time as a succession which as such separates the before and the after. This is beneficial for the sufferer, it cures by separating him “from his pain or from the object of his pain” (Sartre 1956; p. 135). At this point, Sartre’s approach reminds of Buytendijk’s description of the human excentric positionality, that allows him to distance himself from his pain, thereby enabling him to reflect and analyse it (Buytendijk 1948).

On the other hand, time also connects two moments: “If, then, time is separation, it is at least a separation of a special type—a division which reunites” (Sartre 1956; p. 135). Due to this (re-)union provided by time, events become cohesive and interrelated. This is also true for an illness with its different stages, moments of aggravation and respite. As such, Sartre defines illness as “a reality which has its own time” (Sartre 1956; pp. 276-7). The illness cannot be separated into alternating, distinct segments of presence vs. absence of illness and pain; rather it is structured by pain just as a melody that encompasses crescendo and decrescendo: “A pain which is given in twinges followed by lulls is not apprehended by reflection as the pure alteration of painful and non-painful consciousnesses. For organizing reflection the brief respites are a part of the illness just as silences are a part of a melody.” (Sartre 1956; p. 277). Beyond imparting structure and “melody” to the illness, it is pain that constitutes it: “… each concrete pain is like a note in a melody: it is at once the whole melody and a “moment” in the melody. Across each pain I apprehend the entire illness and yet it transcends them all, for it is the synthetic totality of all the pains, …” (Sartre 1956; p. 277).

Svenaeus, in his interpretation of Sartre, emphasizes an important aspect: pain is not primarily an object to be studied scientifically, rather it is a phenomenon to be lived: “Pain is not primarily objectified and reflected upon, but rather lived as a melodic style of human experience“ (Svenaeus 2015; p. 113). The analogy of pain as a melody touches upon the dominating effect of illness and pain might have upon the individual’s life, the all absorbing qualities they display, their alienating effects on the lived body.

In this context, Svenaeus points towards Merleau-Pontys work on the phenomenology of perception, which he considers more profound on the theme of bodily alienation than Sartre (Svenaeus 2015; pp. 114-5). While Merleau-Ponty does not develop a distinct phenomenology of pain, this is accomplished by Christian Grüny, who also includes passages on temporality, mainly drawing on Merleau-Ponty’s phenomenology and on Thomas Fuchs’ enactivist approach. Grüny takes up the idea of detachment, excentricity, or – in its negative perspective – alienation of the sufferer from her pain and interprets it on the backdrop of temporality. While it captures the subject in the present, the sufferer projects herself into a future that she anticipates as pain-free, an approach leading to “extreme forms of self-division” (Grüny 2004; p. 155; translation *JW*). However, this anticipation is tainted with worries about the potential recurrence or even intensification of the pain, the future is coloured with negative emotions (Grüny 2004; p. 174). It is in this context that Grüny uses the term “Bruch” (break, rift): the individual becomes unable to plan for and act into a future potentially marred by unbearable pain. She is trapped in a present filled with disappointment about the continuing absence of the hoped-for state of pain relief (Grüny 2004; pp. 174-5).

After a brief depiction of the neuroscientific view on temporality, I will attempt to elaborate on Grüny’s Merleau-Ponty-based theory of the mutual interaction between timing, temporality, and pain. In particular, the concept of the ‘break’ will be detailed, drawing parallels to Merleau-Pontys concept of the lived and the objective body.

### Temporality – the perspective of neuroscience

The neurobiology of time perception has seen an immense increase of knowledge during the last two decades. As such, it is too complex to present in all details in this article (for an excellent review see Robbe D., Neurosci Biobehav Rev. 2023 Oct;153:105312). I will therefore focus on those aspects relevant for the phenomenological discussion of timing and temporality.

So-called ‘time cells’ are neurons mainly located within the hippocampus – a part of the brain associated with the storage of episodic memory. They are credited with coding the temporal aspects of memories by being activated at specific time-points of an experience (Umbach et al. 2020; p. 1). We can distinguish a ‘microtime’ from a ‘macrotime’ (Mau et al. 2018; p. 2; Alexander et al. 2020; p. 3). Time periods of 10 to 20 s (microtime) are coded by groups of neurons activated successively in the same order, i.e. every neuron ‘fires’ at a reproducible instant of an experience or activity lasting multiple seconds. In contrast, modifications of these neuronal groups can be seen over longer time frames (hours to days; macrotime). While a subgroup of the neuronal network keeps being activated unvariedly, groups of cells will be added or subtracted. Hence, new pieces of memory will be integrated with the immediately preceding context retained. The reproducibility of the activation of neuronal subgroups may also aid in making predictions about the future (Mau et al. 2018; p. 7).

These neural networks do not code for temporality in isolation, but rather within a context of spatial and motional information such as the place of a specific event or the direction and velocity of a movement performed during a given moment (Alexander et al. 2020; p. 1). This neuroscientific perspective on the tight interconnection of temporo-spatial information parallels the philosophical view on the complementarity of the objective vs. lived body and the posit of a subjective, experienced vs. an objective, measured time. Some hippocampal neurons code for temporal as well as for spatial information, allowing for the integration of these qualities on a cellular level (Kraus et al. 2015; p. 19). The egocentric memory concerning localization in space, direction and velocity of movement is closely linked with the intrinsic perception of time. This allows for experiencing a sequence of events as an inherently logical succession and memorize them as a coherent spatiotemporal trajectory rather than loose fragments (Alexander et al. 2020; p. 1; Gallagher and Varela 2003, 36 − 7). This tight interrelation of corporal spatiality and temporality suggests that modifications of the lived body may influence the perception of time. Taking chronic back pain as an example: every distance one must cover will appear more laborious and longer (concerning time and distance) retro- as well as prospectively.

### Pain induces desynchronization of subjective and objective time

The ambiguous character of timing vs. temporality parallels the alternating dominance of the phenomenological lived and the objective body. Just as the individual may become totalized and isolated from her surrounding social and material environment when the focus shifts away from the lived, perceiving unto her objective, biological, tangible body, accentuation of objective time initiates the same process (Fuchs 2006; pp. 196-7). If acting within the implicit, intrinsic time can be interpreted as existing obliviously in the present instant, of nimbly drifting with an unconscious flow of time, the explicit, extrinsic time may be considered conscious, fragmented, and cumbersome. The change from one mode of temporal perception to the other can be provoked by experiences of impediments, limits, and obstacles. And just as the spatiality of the objective body may predominate in moments of pain, the unexpected, undesired may lead to a breach in time^4^:

In contrast to implicit time, then, an explicit experience of time is deemed secondary. It appears on the scene when actions are either interrupted or fail, that is, when an experience or perception seems to be incomplete or fragmentary, or something unexpected happens. In this sense, one could argue that explicit time is motivated by a deviation from the concordant or normal structure of experience (Wehrle 2020; p. 507).

The loss of the sense of temporal continuity caused by chronic pain or other diseases may not only alter the perception of the past, but also project into the future. For Carel, this process forms part of the ‚bodily doubt’ (Carel 2018, 97–101). Due to the interruption of the accustomed flow of everyday activities, the discontinuity of perception and the loss of goal-oriented action resulting thereof, synthesis of present and future fails. The body in pain is an inwardly oriented, non-projecting, dislocated body – much more a material object than an intentional subject. It persists in the present that denies it a future free from suffering, that does not allow for free development (Bozzaro et al. 2015, 18 − 9). Pain, at least once it has become chronic and has extinguished any positive future perspective, becomes a totalizing, isolating experience, a dominance of the present, a lack of future perspective, an abundance of the past (Wandruszka, 2015, 77 − 9).

Just as the objective body displaces the lived body, the ‘Not-any-more’ and ‘Not-yet’ of past and future shroud the lived moment. The individual suffering from pain is deprived of the jauntily flowing, implicit time. This illustrates the parallelism of the transparent lived body and implicit temporality: „Lived time may be regarded as a function of the lived body, …” (Fuchs 2006; p. 196). The lived, implicit time furthers the synchronization with one’s environment, which is disrupted by its explication. Fuchs distinguishes two tendencies: the acceleration versus the deceleration of the internal temporality as compared to the external timing. He describes melancholia with its painfully slow passing of time as an example of decelerated temporality: „Desynchronization, explication of time, and corporalization of the body are joint characteristics of the melancholic state” (Fuchs 2006; p. 196).

This triad can also be postulated for the state of chronic pain and hence stresses its phenomenological similarity to depression. In extreme cases, the implicit temporality may halt completely. The totalizing feature of pain (Maio, 2015, 170-3) claiming all attention, rendering it impossible to distance oneself from one’s aching body, may bring all sense of time to an end:

In such cases of intense pain, we are not only unable to distance ourselves from our body, …, we also lose every sense of time (and space), because neither do we experience change and movement on the level of implicit time or intentional temporality, nor can we refer to our body as a temporal or perceived object anymore (Wehrle 2020; p. 516).

This development ultimately leads to the loss of all intentionality, of the capacity to relate to the individuals and objects making up one’s environment. Intentionality can only be realized by transcending the very instant – it is made impossible by being caught up in the moment (Olivier 2007; p. 105).

### Pain fractures implicit temporality

Just as the intrinsic, subjective – or personal (Merleau-Ponty 2013; p. 86) – experience of time may be extended or accelerated, it is also susceptible for fissures and interferences. While the objective, impersonal time progresses constantly, subjective temporality is determined by the specifics of the individual and her experience:

Impersonal time continues to flow, but personal time is arrested. …Insofar as I inhabit a “physical world,” where consistent “stimuli” and typical situations are discovered – and not merely the historical world in which situations are never comparable – my life is made up of rhythms that do not have their reason in what I have chosen to be, but rather have their condition in the banal milieu that surrounds me (Merleau-Ponty 2013; p. 86).

With this in mind, Dietrich describes pain as a transgression of temporal limits. Pain disrupts the habitual rhythm of life. It imposes on the individual its very own throbbing, pulsating, surging, and fading rhythm (Dietrich 2015, 364-6). It also evokes a break within the intrinsic temporality: it takes one’s breath away, stalls one’s movements, silences one’s communication – pain governs the structure of time (Dietrich 2015, 364).

The alteration of temporality also evokes a break in the individual biography. The prereflexive experience of time is replaced by a conscious perception of temporal structures. Pain bipartitions life into a ‘before’ and an ‘after’. Rather than indicating a problem, pain becomes the problem. The sole permanence of pain, the forced acknowledgement that it will last, makes it unbearable. The individual in pain has been expelled from her normality, making the return the most pressing goal (Grüny 2004; p. 172). Pain always goes along with fearing that it may be unbearable in its limitlessness (Schiltenwolf, 2015, 240; Gadamer 2021; p. 102).

In this light, Fuchs interprets the transition from an implicit to an explicit experience of time as a yearning for a moment that has not yet materialized or the morning of an epoch that has vanished (Fuchs 2006; p. 195). Pain is a prime example of an event that may induce such a break. The chronic pain patient will yearn for a future without pain and regretfully remember the time preceding the onset of her symptoms, even if the memory of being pain-free has probably faded to such an extent as to render reliving, re-embodying this sensation impossible: „participants^5^ continued to refer to ‚then’ and ‚now’ with little future orientation…. … overwhelming sense of loss of a previous, valued self and rejection of a present self” (Snelgrove, 2017, 139 − 40).

This disruption caused by chronic disease has also been described in the clinical context of polyarthritis (Bury 1982, 167–182). Patients experience a biographical break as an alteration of the obvious, of the conceptions of life that have so far been taken for granted. This intrudes on the concept of Self, on the perception of one’s biography, the trajectory of one’s life and illustrates once more the overwhelming influence the lived moment exerts on past and future.

### Pain and the double horizon of temporality

Just as the double horizon of temporality imparts continuity and cohesion to the subjective time, this same horizon – the future that I expect as well as the past that reverberates in the lived moment – will be modified by the present in a circular interaction. For this, the state of our lived body plays a major role, as it „is the means of our communication with both time and space” (Merleau-Ponty 2013; p. 187). Hence, remembering the past is not solely a mental feat, but inherently physical: „The role of the body in memory can only be understood if memory is not the constituting consciousness of the past, but rather an effort to reopen time beginning from the implications of the present…“ (Merleau-Ponty 2013; p. 187). This physical memory will always be hazy: a pain recalled cannot equal a pain acutely experienced: „Just as it is necessarily “here,” the body necessarily exists “now”; it can never become “past”” ((Merleau-Ponty 2013; p. 141; Förster 2008, 28 − 9). However, pain will modify the selection and perception of those moments that are lifted from the horizon of the past into the present. The present state-of-being induces the physical realization of a bygone experience and thereby tints the past in the light of the present inner state.

Pain does not only shape the mental and physical projections of the extended moment and the past, but also future perceptions (Fuchs 2008; p. 319). Painful experiences and our interpretation thereof become imprinted in our pain memory and modify our sensations. Reorganization of neuronal networks leads to a persistence of the painful sensation even in the absence of the stimulus. It can now even be elicited by imagining a trigger movement of the affected extremity or by an optic illusion of touch of the painful area (Moseley & Flor, 2012, 647). Furthermore, functional imaging has established cortical reorganization in patients suffering from chronic pain, including a shift of pain representation from sensory areas to those connected with emotion and affect (McCarberg & Peppin, 2019, 2421). This shift reinforces emotionalization of pain, altering the perception of future experiences.

A study on the temporal perspective in chronic pain patients constitutes a specific example of this effect. Dany et al surveyed 264 patients that presented at a specialized pain clinic for the first time (Dany et al. 2016, 295–308). Among others, ‚time perspective’ (TP)^6^, socioeconomic status and ‚pain beliefs’^7^, pain characteristics and demographic data were analyzed. A significant correlation was found between socioeconomic deprivation, pain constancy, self-blame and the time perspectives ‚past-negative’ and ‚future’ with depression and / or anxiety. This may reflect the lack of control patients feel when experiencing their pain as enduring (Dany et al. 2016; p. 303). The time perspective ‚past-negative’ may result from constantly pondering a past experienced as hurtful and the regret to have lived such painful events (Dany et al. 2016; pp. 303-5). This focus on a grievous past on the other hand will affect current intrapsychic perception and increase pain sensitivity, promote a vicious cycle and elicit further disturbing thought patterns (Simon et al., 2022).

## Conclusion and outlook

On the backdrop of the phenomenology of the lived body in Merleau-Pontys interpretation and the enactivism represented for example by Gallagher and Fuchs, two dominant aspects of the interaction of pain and temporality have been elucidated. Pain desynchronizes and fractures subjective time, inducing isolation of the individual. The effortless flow of consciousness gives way to a fragmented, measurable extrinsic or objective time. Pain becomes totalizing, trapping the sufferer in the present, imposing its very own rhythm on her life. It extinguishes her will to project into a future which – just as the past – is tinged by a present filled with pain.

This work contributes to the phenomenological understanding of pain and temporality by drawing on Merleau-Ponty’s concept of the lived body and lived time and applying it to the study of temporality and pain. Merleau-Ponty himself did not develop a detailed theory on this topic. This is partially remedied by Christian Grüny. His ideas on temporality and pain are pursued in this article and complemented by contemporary enactivist views. Furthermore, added value of this work lies in the demonstration of parallels between the phenomenological and the neuroscientific approach.

While empirical sciences fall into a different category from Husserlian temporalization, some neuroscientific data on time perception recall his concept of retention and protention. This has been exemplified by a reference to hippocampal time cells that are activated in a way as to distinguish micro- from macrotime. While microtime may represent the natural science analogon of the present – the lived – moment, macrotime seems to (partially) explain the ability to experience time as a continuum rather than a series of fractured moments. Time cells multitask: they jointly code temporal and spatial information. This very much reminds of Merleau-Ponty’s concept of the lived body as a reference in space and time and supports the approach of this article – to develop a phenomenological concept of pain and temporality drawing on a theory on the lived body and its spatiality. Further parallels between phenomenology and the neurosciences have been pointed out at the level of functional imaging data that show reorganisation of cortical networks probably associated with pain memory and the emotional attributes of painful experiences (Moseley & Flor, 2012; McCarberg & Peppin, 2019). The practical relevance of our conclusions have been illustrated by clinical studies on patients with polyarthritis and other pain syndromes (Bury 1982; Dany et al. 2016).

I conclude that just as spatial perception, temporality results from the reciprocal interaction of the subject and her objective environment, enabling the experience of one’s own existence as continuous and the creation of a meaningful ‘ecological time-niche’. Intrinsic experience of time – or lived time – is closely entwined with concurrent spatial circumstances and thus with the lived body. As the latter is undoubtedly affected by pain, we may also postulate a mutual influence of temporal perception and chronic pain. The isolating, totalizing experience that the loss of transparency of the lived body entails, is paralleled and enhanced by the explication and desynchronization of temporality.

Pain has seemingly contradictory tendencies on time perception: it contracts time, the individual remains suspended in the moment. On the other hand, pain extends the moment by casting its shadows on the past as well as on the future (Olivier 2007; p. 116). The natural course of intrinsic temporality, its synchronization with the objective time is fractured, the individual is expelled from her world and thrown back on herself. Pain, perception, and time relate in a circular way (Olivier 2007; p. 118; 122-3). Pain alters the perception of time, for instance by inducing a fixation on the current moment. This modification of temporality invariably influences all other sensations – including pain itself. Hence, pain may be alleviated not only by direct analgesic intervention, but also by changing the perspective on time and interrupting its monotony: „Such change might not alleviate or stop the pain, but it certainly removes its captivity” (Olivier 2007; p. 123).

This change of perspective may be induced by actively shaping the perception of the future perspective:

The ‘future’ perspective has a beneficial effect on depression. Future orientation can constitute an interesting way to analyze adaptation to chronic pain because it is associated to proactive coping (…). The future-oriented person always has an eye toward consequences, contingencies, and probable outcomes of present decisions and actions. Acceptance, in turn, includes awareness of pain with continuation of desired activities and without struggling for control over pain (…) (Dany et al. 2016; p. 305).

In this light, approaches of cognitive and behavioral therapy may be used to shift one’s focus from continually reliving negative past experiences to adopting a more accepting attitude. Particularly mindfulness training and cognitive-behavioural intervention (CBI) to challenge incorrect beliefs that pain is never ending and to build acceptance for a life that is different from that the individual hoped for has been proven valuable in this context. In terms of a “top-down reorganization”, activation of descending inhibitory tracts and central restructuring – particularly of the insular and cingulate cortex – have been proposed as anatomical and physiological correlates of therapeutic efficiency (de Ridder et al. 2021; Su et al. 2016). Existential psychotherapy may pose an alternative to behavioral therapy. In the tradition of Viktor Frankl and Irvin Yalom, this technique focuses on enabling the individual to make autonomous choices, even in the face of – and in the end empowered by – her existential vulnerability and integrate the perception of pain into a meaningful life: „A person’s being is continually becoming, but this becoming is increasingly his or her own doing. Through their decisions and actions, human persons shape their own development“ (Fuchs 2020; Frankl 2007; Yalom 2010).

## Footnotes

1 the authors included primary and secondary costs of illness.

2 See Dietl 2011, p. 17 (https://portal.dimdi.de/de/hta/hta_berichte/hta301_bericht_de.pdf); the DIMDI (German Institute of Medical Information and Informatics) formed part of the German Ministry of Health.

3 The author relates her experiences in the context of dyspnoea caused by a chronic progressive pulmonary disease.

4 Moments of disruption are not the only instances of switching to explicit time, as we enter this mode every time we consciously plan or schedule for the future.

5 „participants“ are patients suffering from chronic back pain that have been subjected to phenomenological interviews.

6 TP was tested via the Zimbardo Time Perspective Inventory (Zimbardo & Boyd, 1999) containing 54 items on five subscales: ‘past-negative’ (aversive attitude towards the past), ‘past-positive’ (positive construction of the past), ‘present-hedonistic’ (orientation towards pleasure and enjoyment in the present), ‘present-fatalistic’ (hopeless, nihilistic attitude towards life), and ‘future’ (future goals are planned and achieved).

7 ‚Pain beliefs‘ are patients‘ perceptions of diverse pain characteristics, such as the belief if the pain will be permanent or if it will be constant rather than intermittend. The authors use the Perceptions Inventory (Williams & Thorn, 1989).
